# Impact of manual sepsis screening in hospitalized adult patients: A systematic review

**DOI:** 10.1002/jhm.70284

**Published:** 2026-02-15

**Authors:** Rachel K. Hechtman, Natasha Garamani, Yezen R. Anabtawi, Amanda G. Rivas, Bailey Antonowicz, Anna K. Barker, Tami Garcia, Rosalie Mulcahy, Patricia J. Posa, Michael W. Sjoding, Hallie C. Prescott

**Affiliations:** ^1^ Department of Medicine University of Michigan Ann Arbor Michigan USA; ^2^ Michigan State University College of Osteopathic Medicine East Lansing Michigan USA; ^3^ VA Center for Clinical Management Research Ann Arbor Michigan USA

## Abstract

**Background:**

Manual sepsis screening, which includes bedside clinical assessment, is widely used in emergency departments and hospital wards and may improve early recognition and treatment.

**Objectives:**

To synthesize evidence on the impact of manual sepsis screening on sepsis‐related processes of care and mortality.

**Methods:**

We performed a systematic search of MEDLINE, Cochrane, Embase, and CINAHL for original research published between January 1, 2000 and July 31, 2024. Search terms addressed sepsis, screening, and hospital settings (emergency department, ward, intensive care unit). We included full‐text studies evaluating the impact of manual screening on sepsis‐related processes of care or mortality. Two reviewers screened studies, and risk of bias was assessed using the ROBINS‐I tool.

**Results:**

Of 10,469 studies identified, 17 met inclusion criteria. Studies were conducted in nine countries; most were single‐center (*n* = 15), used pre‐post designs (*n* = 15), and were conducted before 2016 (*n* = 11). Seven of 10 studies reported improvement in at least one process‐of‐care outcome after implementation. Six of 13 studies reported statistically significant mortality reductions; three additional studies reported ≥5% absolute mortality improvement without statistical testing or significance. Overall conclusions were limited by high risk of bias, primarily due to confounding.

**Conclusions:**

Evidence supporting manual sepsis screening is low quality and may be outdated given evolving sepsis recognition and care. Rigorous contemporary studies are needed to guide adoption and implementation decisions.

## Visual Abstract

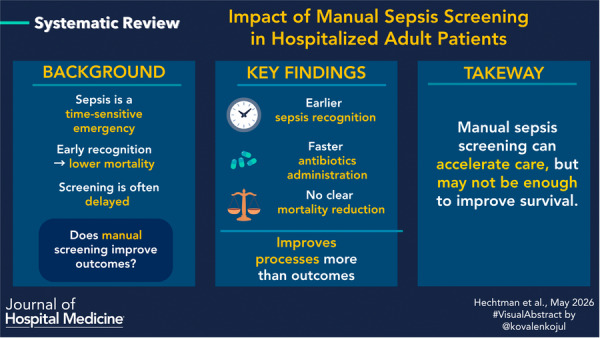

## INTRODUCTION

Sepsis is a leading cause of in‐hospital mortality, and early identification and treatment are critical for improving patient outcomes.^1^, ^2^, ^3^ To identify patients early in their disease course, the 2021 Surviving Sepsis Campaign Guidelines strongly recommend screening at‐risk patients for sepsis.^4^ However, the guidelines do not specify how patients should be screened. The most common strategies are automated screening in the electronic health record (EHR) and manual screening, which involves bedside assessment by the person doing the screening. There are several potential drawbacks of automated screening tools embedded within the EHR,^5^ including poor model performance,^6^, ^7^ alarm fatigue,^8^ limited accessibility in lower‐resourced settings,^9^ and mixed evidence on effectiveness for improving sepsis‐related processes and outcomes.^10^, ^11^ As data to support automated sepsis screening, with or without artificial intelligence, has not consistently demonstrated improvement in sepsis‐related processes of care and patient outcomes, hospitals may reach for a “back‐to‐basics” approach. Manual sepsis screening, defined as any sepsis screening process that involves clinician bedside assessment by the person doing the screening (often completed by a nurse), offers an alternative approach that is accessible across care settings.

Manual screening is already widely implemented, alone or in parallel with automated screening,^12^, ^13^, ^14^ yet the quality of evidence to support the use of manual screening is unknown.^15^, ^16^, ^17^, ^18^ The impact of sepsis screening is a priority of the Agency for Healthcare Research and Quality's (AHRQ) Evidence‐based Practice Center Program and AHRQ has published two reviews evaluating systems used to improve sepsis recognition and outcomes.^19^, ^20^ The reviews found wide variability in the performance of sepsis screening tools and moderate evidence for improvement in sepsis processes of care.^20^ They also found that machine learning and automated sepsis risk calculators may reduce mortality, though results were not all statistically significant.^19^ However, these reviews and others focus on test characteristics of screening tools and randomized controlled trials,^21^, ^22^ not on a comprehensive assessment of mortality outcomes with manual screening including the many pre‐post studies, which is essential information for hospitals and patient safety programs considering putting resources toward implementing manual screening.

To inform health systems that are considering implementing manual sepsis screening in the emergency department (ED), hospital wards, or intensive care units (ICU), we conducted a systematic review that sought to evaluate the available evidence on the impact of manual screening on sepsis‐related processes of care (e.g., antibiotic delivery, fluid resuscitation) and patient mortality.

## METHODS

### Search strategy and study selection

We conducted the search, screening, and data extraction as outlined in our pre‐registered protocol.^23^ An experienced information specialist assisted with development of the search strategy. We searched MEDLINE (OVID), Cochrane (Wiley), Embase (Elsevier), and CINAHL (EBSCO) for original research studies published from January 1, 2000 through July 31, 2024 using search terms related to sepsis and screening in ED, hospital, and ICU settings. We restricted the search to human studies available in English. MEDLINE searches were adapted to other databases using the Polyglot Search Translator^24^ (complete search strategy available in the supplement). The citation lists of included studies were hand‐searched to identify additional studies. Studies were screened by two reviewers in each step, and disagreements were resolved through discussion. The study follows the Preferred Reporting Items for Systematic Reviews and Meta‐Analyses (PRISMA) guidelines,^25^ as well as the Cochrane guidelines for systematic reviews.^26^

Following the initial search, duplicates were removed and five reviewers (R.K.H., N.G., Y.A., A.R., and B.A.) independently performed title and abstract screening using eligibility criteria outlined below. Full text screening was then performed by three reviewers (R.K.H., N.G., and Y.A.). During title and abstract screening, reviewers had 83.2% agreement (mean pairwise kappa, *k* = 0.43). During full‐text review, three reviewers had an agreement of 88.0% with mean pairwise kappa statistic, *k* = 0.66, indicating substantial agreement.

### Inclusion/exclusion criteria

We aimed to include studies that evaluated the impact of manual screening for sepsis, clinical instability, or clinical deterioration on sepsis processes of care and outcomes in hospitalized adults. During title and abstract screening, we included studies that evaluated the impact of screening alone, or multicomponent interventions in which screening was reported as a component of the intervention in the title or abstract. We included both peer‐reviewed journal articles and gray literature (conference abstracts and theses). However, during full‐text review, we included only studies with full texts available. If multiple articles reported similar outcomes on the same study, we contacted the authors and confirmed that the article with the latest publication date should be included because it had the most comprehensive and up‐to‐date results.

### Risk of bias assessment

Risk of bias was assessed using the ROBINS‐I V2 (Risk Of Bias in Non‐randomized Studies—in Interventions) tool.^27^ One reviewer assessed seven domains and an overall risk of bias for each study. No studies were excluded from the review based on methodological quality to maintain a comprehensive analysis.

### Data extraction and synthesis

The data extraction tool was designed based on the study aims and protocol. Data extraction was performed by two reviewers (R.K.H. and Y.A.). Information extracted from each article included author, year, country, study design, setting, and population, details on the screening tool used and how it was implemented, and the gold standard used to identify true sepsis cases. Missing data were recorded as not reported (NR). The primary outcomes for the review were sepsis‐related process measures (including collection of lactate and blood cultures, administration of antibiotics, and IV fluids) and mortality. Study outcomes were presented in three categories: statistically significant difference; clinically significant difference (defined as ≥5% absolute difference) that did not meet threshold for statistical significance due to small sample size or statistical significance was not reported; and no difference. Subgroups by study location (ED, hospital ward, and ICU) were evaluated in secondary analysis. Secondary outcomes extracted were performance of the screening tool and metrics on screening adherence. The results were synthesized through narrative review.

## RESULTS

### Search results

**Figure 1:**
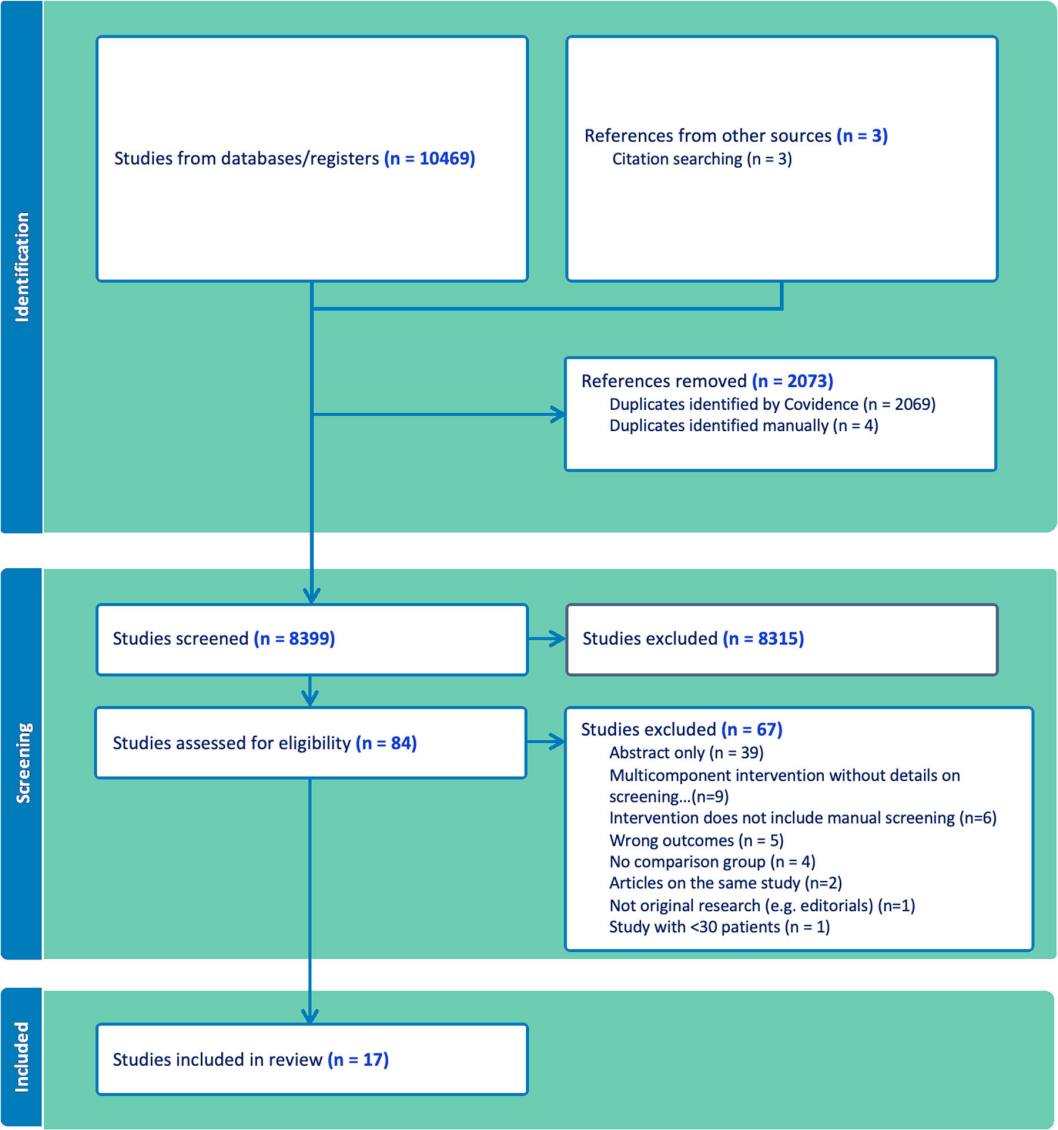
Study flow diagram.

The search identified 10,469 records and an additional three studies were identified through citation searching. These were exported into Covidence where 2073 duplicates were removed. The resulting 8399 records were screened by their title and abstract. Subsequently, 84 abstracts were considered potentially relevant and screened for full‐text articles. Thirty‐nine of these were excluded because they were abstracts without an associated full text, 26 were excluded due to exclusion criteria pre‐specified in the protocol, and two were excluded as they reported results from the same study as an included article. A resulting 17 studies were included for analysis. A PRISMA flow diagram was generated to demonstrate the process (Figure 1).

### Study characteristics

Of the 17 included studies, 16 were published in peer‐reviewed journals and 1 was a doctoral dissertation. Most studies were single center (*n* = 15), pre‐post intervention studies (*n* = 15). Studies were performed in nine different countries, most commonly the United States (*n* = 8) and Canada (*n* = 2) (Table 1). Eleven studies were conducted prior to the 2016 updated definition for sepsis and all were performed prior to the COVID‐19 pandemic. The studies examined sepsis screening in EDs (*n* = 9),^28^, ^29^, ^30^, ^31^, ^32^, ^33^, ^34^, ^35^, ^36^ hospital wards (*n* = 4),^37^, ^38^, ^39^, ^40^ ICUs (*n* = 3)^41^, ^42^, ^43^ and on screening implemented across all three locations (*n* = 1).^44^ Among studies that reported total sample size used in the analysis (*n* = 15) the median sample size was 355 patients (interquartile range 156–756).

**Table 1:** Study details.

Author, year	Country	Setting	Study design	Sample size
Emergency department (ED)				
Bader, 2020	Jordan	Nonprofit cancer center	Pre‐post	168 (85 intervention, 83 control)
Hart, 2017	USA	Academic medical center	Pre‐post	130 (67 intervention, 64 control)
Idrees, 2016	Australia	Urban general hospital, ICU patients admitted from ED or w/in 48 h of ward admission	Pre‐post	90 (45 intervention, 55 control)
McDonald, 2018	Canada	Teaching hospital	Pre‐post	616 (270 intervention, 346 control)
Patocka, 2014	Canada	Teaching hospital	Pre‐post	355 (170 intervention, 185 control)
Shah, 2018	USA	Academic medical center	Pre‐post	115 (57 intervention, 58 control)
Song, 2019	South Korea	Academic medical center	Pre‐post	631 (315 intervention, 316 control)
Suttapanit, 2022	Thailand	Academic medical center	Pre‐post	479 (153 intervention, 153 control)
Tedesco, 2017	USA	Community hospital	Pre‐post	Not reported
Hospital ward				
Alberto, 2020	Argentina	Tertiary referral hospital	Interrupted time series	145 (64 intervention, 81 control)
Jones, 2015	USA	Teaching hospital	Pre‐post	15,353 (10,479 intervention, 4874 control)
Roney, 2019	USA	Acute care hospital	Pre‐post	Not reported
Torsvik, 2016	Norway	Community hospital	Pre‐post	881 (409 intervention, 472 control)
Intensive care unit (ICU)				
Croft, 2014	USA	Academic medical center SICU	A/B testing (paper vs. EHR‐based screen)	184 (65 paper screening, 119 EHR‐based screening)
Moore, 2009	USA	Academic medical center SICU	Pre‐post	920 (920 intervention, control sample size not reported)
Rincon, 2011	USA	Hospital system including ICU beds across 10 hospitals monitored by telemedicine nurses	Pre‐post	5437 (5437 intervention, control sample size not reported)
ED, hospital ward, and ICU				
Westphal, 2011	Brazil	2 hospitals “with average to advanced levels of medical care”	Pre‐post	217 (115 intervention, 102 control)

### Interventions

Screening was performed by nurses or nurse technicians in 16 studies (94%) studies and was completed on paper forms in eight studies (Table 2). Screening in the ED was most often performed at triage (*n* = 7) and screening in the hospital wards or ICU was most often performed during each 8–12 h nursing shift (*n* = 4) or with each assessment of vitals (*n* = 2). Eleven studies assessed multicomponent interventions, where screening was implemented concurrently with a new sepsis protocol (*n* = 8), with visual flags in the EHR to indicate the patient screened positive for sepsis (*n* = 3), with new nurse‐initiated sepsis workup (*n* = 2), and/or with rapid response team activation for a positive screen (*n* = 2). In six studies, sepsis screening was the only intervention (a sepsis protocol was in place prior to the study). In all studies, a positive screen triggered the screener to notify a physician and/or other provider (nurse practitioner, physician's assistant, or charge nurse).

**Table 2:** Intervention protocols and comparison group used.

Author, year	Screening provider and timing	Screening format	Concurrent interventions	Comparator description
Emergency department (ED)				
Bader, 2020	Research team at triage	Paper	Sepsis protocol	Pre‐implementation
Hart, 2017	Nurse at triage	Electronic	None	Pre‐implementation (sepsis protocol in place)
Idrees, 2016	Nurse at triage	NR	Sepsis protocol	Pre‐implementation
McDonald, 2018	Nurse at triage	Paper	Electronic health record (EHR) sepsis indicator, nurse can order sepsis workup (labs and CXR)	Pre‐implementation (sepsis protocol in place)
Patocka, 2014	Nurse at triage	NR	Nurse can initiate sepsis workup if hypotensive	Pre‐implementation (sepsis protocol in place)
Shah, 2018	Nurse at triage	Electronic	EHR sepsis indicator	Pre‐implementation (sepsis protocol in place)
Song, 2019	Nurse at triage	Electronic	Sepsis protocol, EHR sepsis indicator, EHR alert to physician	Pre‐implementation
Suttapanit 2022	Nurse at triage	Paper	None	qSOFA was used to screen prior to study implementing REW score (sepsis protocol in place)
Tedesco, 2017	ED nurse “when sepsis was suspected”	Paper	Sepsis protocol, sepsis response team called	Pre‐implementation
Hospital ward				
Alberto, 2020	Nurse at admission, Q8 hour shift, and for clinical change	Paper	Sepsis protocol	Pre‐implementation
Jones, 2015	Nurse at admission and Q12 hour shift	Electronic	Sepsis protocol	Pre‐implementation
Roney, 2019	Nurse (frequency NR)	Paper	Sepsis protocol, rapid response team called if high score	Pre‐implementation
Torsvik, 2016	Nurse Q4 hour if suspected infection	NR	Sepsis protocol	Pre‐implementation
Intensive care unit (ICU)				
Croft, 2014	Nurse Q4 hour with vitals	Paper, then electronic	None	Paper versus electronic screening tool(sepsis protocol in place)
Moore, 2009	Nurse Q12 hour shift	NR	None	Pre‐implementation + control ICU (sepsis protocol in place)
Rincon, 2011	Tele‐ICU nurses at ICU admission and Q12 hour shift	Electronic	None	Pre‐implementation (sepsis protocol in place)
ED, hospital ward, and ICU				
Westphal, 2011	Nurse technician (frequency NR)	Paper	None	Pre‐implementation (sepsis protocol in place)

*Note*: EHR sepsis indicator = electronic health record indicator (banner, flag, etc) to visually alert clinicians that the patent has/may have sepsis; Q4/Q8/Q12 h = every 4/8/12 h.

Abbreviations: CXR, chest x‐ray; MEWS, Modified Early Warning score; NR, not reported; qSOFA, quick Sequential Organ Failure Assessment; SIRS, Systemic Inflammatory Response syndrome.

The most common screening tools used were Systemic Inflammatory Response Syndrome (SIRS) (*n* = 4), quick Sequential Organ Failure Assessment (qSOFA) (n = 2), and Modified Early Warning Score (MEWS) (*n* = 2), though most studies used a combination of elements from these tools or a custom screening tool (Supporting Information S1: Table S1). All studies used screening tools that required bedside assessment, most commonly for altered mental status (*n* = 13), increased oxygen requirement (*n* = 8), decreased urine output (*n* = 6), or evaluation for suspected infection (*n* = 3).

### Identification of sepsis cases

The reference standard definitions used to identify sepsis cases for analysis varied. Of the 14 studies that reported how they identified sepsis cases, seven studies used International Consensus Definitions for sepsis: three used the sepsis‐3 definition,^45^ and four used the prior definitions for sepsis, severe sepsis, and septic shock^46^ (Supporting Information S1: Table S2). In studies using consensus definitions, sepsis was confirmed via single‐physician retrospective chart review in three studies, via prospective screening and consensus determination in one study, and was unreported in one study. Six studies used International Classification of Disease (ICD) codes to identify sepsis cases (ICD‐9 *n* = 4 vs. ICD‐10 *n* = 2), and one study analyzed only sepsis cases with a positive blood culture.

### Impact on processes and outcomes

Thirteen studies examined sepsis‐related processes of care, including time to antibiotics (*n* = 11), time to collection of lactate and/or blood cultures (*n* = 5), time to intravenous fluid administration (*n* = 4), and overall compliance with sepsis management bundles (*n* = 4) (Table 3). Overall, seven (53.8%) of these studies found that screening was associated with statistically significant improvement in at least one process measure, while six (46.2%) found no statistical significant difference in any processes of care assessed. Of the 11 studies that examined antibiotic timing, six reported statistically significant improvement and five reported no improvement with the intervention (Supporting Information S1: Table S3).

**Table 3:** Outcomes: Processes of care and mortality.

Author, year	Process results details	Mortality results details
Emergency department (ED)		
Bader, 2020	– Time to antibiotics: significant decrease – Proportion receiving antibiotics within 60 min: significant increase – Time to blood draw and IV fluids: decrease	1‐month mortality: significant decrease
Hart, 2017	– Time to antibiotics, lactate, blood cultures: no significant difference	
Idrees, 2016	– Proportion receiving antibiotics within 60 min: significant increase	
McDonald, 2018	– Time to lactate, blood cultures, antibiotics, and fluids: significant decrease	ICU mortality: numeric decrease (28.0%–23.5%), not significant
Patocka, 2014	– Lactate measured: significant increase – Time to antibiotics: significant decrease‐ Time to IV fluids: no significant difference	In‐hospital mortality: numeric decrease (21.6%–15.3%), not significant
Shah, 2018	– Time to antibiotics: significant decrease−3‐h bundle compliance: no significant difference	30‐day mortality: no significant difference
Song, 2019	– Time to antibiotics: no significant difference – Sepsis bundle compliance: significant increase	30‐day all‐cause mortality: significant decrease
Suttapanit, 2022	– Proportion receiving antibiotics within 60 min: no significant difference	28‐day mortality: no significant difference
Tedesco, 2017		Yearly sepsis mortality: significant decrease
Hospital ward		
Alberto, 2020	– Proportion receiving at least 1 bundle element within 48 h: no significant difference – Time to completion of any bundle element: numeric decrease (8 h earlier), not significant	
Jones, 2015		In‐hospital mortality: significant decrease
Roney, 2019		Monthly observed versus expected sepsis‐related mortality: numeric decrease (O:E ratio 1.05–1.58 pre vs. 0.81–0.9 post), significance not reported
Torsvik, 2016	– Probability of receiving appropriate antibiotics within 24 h: no significant difference	30‐day mortality: significant decrease
Intensive care unit (ICU)		
Croft, 2014	– Time to antibiotics: no significant difference	In‐hospital mortality: no significant difference
Moore, 2009		Yearly sepsis‐related mortality: numeric decrease (35%–23%), significance not reported
Rincon, 2011	– Time to antibiotics: significant decrease‐ Lactate measured: significant increase‐ Proportion of hypotensive patients receiving fluid: significant increase	
ED, hospital ward, and ICU		
Westphal, 2011	– 6‐h Bundle compliance: no significant difference	In‐hospital and 28‐day mortality: both significant decrease

*Note*: Green = improvement with intervention, red = no improvement with intervention, yellow = either mixed results or a nonsignificant trend toward improvement.

Thirteen studies reported on mortality, most often 28–30‐day (*n* = 6) or in‐hospital (*n* = 4) mortality. Six studies (46.1%) reported a statistically significant improvement in mortality with the intervention, four (31.8%) reported a clinically significant improvement in mortality (consistent with a ≥5% difference) that did not meet the threshold for statistical significance or statistical significance was not reported, and three (23.1%) reported no improvement (Table 3).

Among studies in EDs, six of nine reported statistically significant improvement in at least one process of care. Of eight ED studies that reported on mortality, four reported statistically improved mortality, two reported clinically significant improvement in mortality (≥5%) that did not meet the threshold for statistical significance or statistical significance was not reported, and two reported no improvement with manual sepsis screening (Supporting Information S1: Table S3). None of the studies in hospital wards reported improvement in any process of care. Three of four studies that reported on mortality found statistically significant improvement, and one reported clinically significant improvement but did not report statistical significance. Among ICU studies, one of three reported improvement in at least one process of care, one reported statistically significant improvement in mortality, one reported clinically significant improvement but did not report statistical significance, and one reported no improvement in mortality with manual sepsis screening.

Six studies reported on secondary outcomes of interest: four reported on adherence to screening^32^, ^37^, ^38^, ^43^ and two reported on test characteristics of the screening tool used^41^, ^42^ (Supporting Information S1: Table S4). The proportion of eligible patients who received screening ranged from 33%^38^ to 92.5%.^37^ One study reported on nursing adherence to the protocol after a positive screen, finding that nurses activated sepsis alerts per‐protocol in 32.8% of positive screening cases.^37^ Of the two studies that reported test characteristics of their screening tool, they found positive predictive values of 29.4% and 80.2%.

### Risk of bias

Most studies reporting on outcomes of manual sepsis screening were pre‐post evaluations of quality improvement efforts, which are subject to bias from multiple sources. The overall risk of bias in 11 of 17 studies was judged to be serious, most often from high risk of bias due to confounding (Supporting Information S1: Table S5). One study was judged to be at low risk of bias.^40^

## DISCUSSION

In this systematic review of manual screening for sepsis, most studies (54%) reported an improvement in at least one process of care after implementing manual screening, 46% reported a statistically significant improvement in mortality, and 31% reported clinically significant improvement in mortality that did not meet the threshold for statistical significance or statistical significance was not reported. Studies were performed in diverse settings globally and used a variety of screening tools, including SIRS, qSOFA, MEWS, and locally adapted tools.

While the majority of studies found improvement in sepsis processes or outcome, most studies were at high risk of bias from several sources. First, most were pre‐post implementation studies that evaluated multicomponent interventions, often as part of a quality improvement effort. Pre‐post studies are at inherently high risk of bias due to confounding, and most studies reported unadjusted outcomes, not accounting for differences that may have occurred due to changes in hospital policy, patient case mix, or baseline improvement in sepsis recognition over time. Second, many studies implemented manual screening as part of a multicomponent intervention, making it impossible to isolate the effect of manual screening specifically. Though similar results were reported in studies that implemented screening alone versus those with multicomponent interventions, there were no studies comparing these scenarios. Third, some studies used different criteria to identify patients for inclusion in their screening and control groups, introducing selection bias. Finally, publication bias may have impacted the available evidence, as nearly half the studies identified in title and abstract screening were conference abstracts that did not have a full peer‐reviewed text. Overall, our review highlights the need for higher quality evidence to help inform hospitals about the benefits of implementing manual screening for sepsis.

Our review extends upon prior studies focused on sepsis recognition. Sepsis screening is recommended by the Surviving Sepsis Campaign guidelines to improve recognition, but there is no recommendation for whether this should be done manually or through automated, EHR‐based screening. As data to support the widespread adoption of EHR‐based screening tools and artificial intelligence (AI)‐prediction models is limited, manual screening remains a reasonable alternative that may even be more accurate due to incorporation of bedside assessment^17^, ^18^, ^47^ and cost effective.^9^ A recently published randomized control trial of over 60,000 patients randomized to screening versus no screening provides an example of a hybrid approach.^48^ Though the screening was done continuously via an automated EHR system (thus excluding it from this review), the screening tool used was qSOFA, which can be performed manually and requires assessment for altered mental status. Patients in the screening group had 15% lower adjusted 90‐day in‐hospital mortality, with a number needed to screen of 206 patients. This positive trial using a tool that can be completed manually suggests screening may not require costly EHR tools or advanced AI models to improve patient care.

In 2000, the AHRQ published a patient safety report reviewing the evidence for manual sepsis identification strategies.^19^, ^20^ It included three studies with manual screening interventions, concluding that there was moderate evidence for process measure improvement with screening.^32^, ^36^, ^43^ However, they found insufficient evidence on the impact of manual screening on patient outcomes. Compared to the search strategies used in the AHRQ reports, we broadened the terms used to describe screening (e.g., to capture studies on sepsis “recognition” or “diagnosis”), included pre‐post studies, and increased the date ranges. This broader search strategy captured more studies relevant to our question on the impact of manual screening, which included several studies that assessed associations between manual sepsis screening and mortality, and more studies evaluating impact on sepsis processes of care.

The integration of AI‐powered prediction models into early sepsis detection workflows has the potential to significantly impact the use of manual sepsis screening. Currently, AI‐based models are costly to implement and have not consistently demonstrated superior test performance compared to traditional screening tools.^8^, ^49^ However, AI‐based prediction and detection models are rapidly evolving, and as their performance improves they may increasingly replace or serve as triggers for manual screening involving bedside assessment. Importantly, both AI‐based and manual screening strategies face challenges with real‐world adherence, due to alert fatigue and override in the case of automated tools^50^ and workload and competing demands on clinician time with manual screening protocols.^32^, ^37^, ^51^ Comparative effectiveness studies should therefore extend beyond test performance to evaluate the impact of manual vs AI‐based screening on patient‐centered outcomes.

Manual sepsis screening is a practice that requires human resources and is most commonly done by nurses. Though screening can be integrated into routine assessments, it requires additional time and attention. This resource allocation concern is underscored by the moderate to low screening adherence rates reported in several studies, despite integrating screening into routine nursing assessments.^32^, ^37^, ^38^, ^43^ Literature comparing the sensitivity of manual versus automated sepsis screening has also found lower sensitivity in manual screening due to high rates of non‐adherence.^51^ For manual sepsis screening to be effective, more work is needed to understand the barriers and facilitators to completing screens and carrying out next steps following a positive screen.

This review must be considered in the context of several limitations. First, it is difficult to draw generalizable conclusions about the effectiveness of manual sepsis screening from the available evidence due to the risk of bias in the included studies (small sample sizes, selection bias, pre‐post study designs, and multicomponent interventions), along with heterogeneity in screening protocols and tools used, and the wide variety of settings. However, our review is the first to synthesize the evidence across countries, hospital settings, and screening tools, providing insights into the breadth and quality of available evidence. It highlights the need for high‐quality studies to isolate the effect of manual sepsis screening on processes of care and outcomes. Second, most studies were performed before the 2016 sepsis definition update, and all were performed prior to the COVID‐19 pandemic, which raises concerns about the generalizability of the findings to current practice settings. Sepsis awareness has increased, and early recognition and treatment has become a priority, with 73% of US hospitals having a sepsis committee.^13^ With increased awareness and concerted efforts to improve quality of care, clinicians may have heightened sensitivity to signs of sepsis, so the addition of formal screening may have differential impacts.

In this systematic review of the impact of manual sepsis screening on sepsis‐related processes of care and outcomes, we found that, though many studies reported an improvement in processes of care and mortality, high risk of bias in the available data limit generalizable conclusions. Health systems considering implementing manual sepsis screening should be aware of limitations to the data surrounding this practice. High‐quality studies are needed to evaluate the effectiveness of manual sepsis screening in current practice, and to identify optimal screening protocols.

## CONFLICT OF INTEREST STATEMENT

The authors declare no conflicts of interest.

## ETHICS STATEMENT

The authors have nothng to report.

## Supporting information

SepScreenRev_R&R_Supplement_2025.10.23.
