# Supplementary material for: Impact of manual sepsis screening in hospitalized adult patients: A systematic review
Source: J Hosp Med. 2026 Feb 15;21(5):539–48. doi: 10.1002/jhm.70284 (PMC13136923; doi:10.1002/jhm.70284)

**Supplement**

**Title**: Does manual sepsis screening in adult hospitalized patients improve management and outcomes of sepsis – a systematic review

**Contents**

| **Item** | **Title** | **Page** |
| --- | --- | --- |
| 1 | Search Strategy | 2 |
| 2 | Table e1: Screening tools used | 5 |
| 3 | Table e1: Methods used to identify sepsis cases | 6 |
| 4 | Table e2: Processes of care and mortality outcomes by study | 7 |
| 5 | Table e3: Outcomes on screening adherence and screening tool test characteristics | 8 |
| 6 | Table e5: ROBINS-I risk of bias assessment | 9 |

**Search Strategy**

Note: the review was originally registered as a rapid review because limitations were made on dates searched (studies published after 2000), language (English), MeSH major topic terms were used for sepsis, and studies were excluded by the search if they included automatic sepsis screening or surveillance. Following the initial search, the study team found the search strategy overly restrictive and removed the terms restricting studies that included automatic sepsis screening or surveillance. This change significantly broadened the search in line with a systematic review rather than a rapid review.

**Date searched:** August 1, 2024

**Date limit:** 2000-present

**Language filter:** English

**Total Results:**  10,469

**Medline:**  4462

**Cochrane:**  292

**CINAHL:**  1655

**Embase:**  4060

**Searches were translated from Medline to other datasets with the assistance of the  Polyglot Search Translator:**Clark JM, Sanders S, Carter M, Honeyman D, Cleo G, Auld Y, Booth D, Condron P, Dalais C, Bateup S, Linthwaite B, May N, Munn J, Ramsay L, Rickett K, Rutter C, Smith A, Sondergeld P, Wallin M, Jones M, Beller E. Improving the translation of search strategies using the Polyglot Search Translator: a randomized controlled trial. J Med Libr Assoc. 2020 Apr;108(2):195–207. doi: 10.5195/jmla.2020.834. Epub 2020 Apr 1. PMCID: [PMC7069833.](https://pubmed.ncbi.nlm.nih.gov/32256231/)

===================================================

**Ovid MEDLINE(R) ALL <1946 to July 31, 2024>**

**Results:**  4462

| Step |  |
| --- | --- |
| 1 | exp *sepsis/ or (septic* or sepsis or septic?emia or "systemic inflammatory response syndrome" or py?emia).tw. |
| 2 | exp mass screening/ or (screen* adj3 (manual* or tool*)).tw. or (identif* or recogni* or diagnos* or screen* or detect* or surveill* or monitor*).ti. |
| 3 | exp Hospitals/ or hospital*.tw. or exp "emergency service, hospital"/ or "emergency department*".tw. or exp intensive care units/ or "intensive care unit*".tw. or ICU.tw. or ((moderate or medium or intermediate) adj5 (unit or units or ward or wards)).tw. |
| 4 | 1 and 2 and 3 |
| 5 | exp animals/ not humans.sh. |
| 6 | 4 not 5 |
| 7 | limit 6 to (english language and yr="2000 -Current") |

**Cochrane (Wiley)**

**Results:** 292

**Note:** Set “Custom Range” filter to 2000-2024

| Step |  |
| --- | --- |
| 1 | [mh sepsis] OR (septic*:ti,ab OR sepsis:ti,ab OR septic?emia:ti,ab OR "systemic inflammatory response syndrome":ti,ab OR py?emia:ti,ab) |
| 2 | [mh "mass screening"] OR (screen*:ti,ab NEAR/3 (manual*:ti,ab OR tool*:ti,ab)) OR (identif*:ti OR recogni*:ti OR diagnos*:ti OR screen*:ti OR detect*:ti OR surveill*:ti OR monitor*:ti) |
| 3 | [mh Hospitals] OR hospital*:ti,ab OR [mh "emergency service, hospital"] OR ("emergency" NEXT department*):ti,ab OR [mh "intensive care units"] OR ("intensive care" NEXT unit*):ti,ab OR ICU:ti,ab OR ((moderate:ti,ab OR medium:ti,ab OR intermediate:ti,ab) NEAR/5 (unit:ti,ab OR units:ti,ab OR ward:ti,ab OR wards:ti,ab)) |
| 4 | (1 and 2 and 3) |
| 5 | [mh animals] NOT [mh humans] |
| 6 | 4 NOT 5 |

**Embase (Elsevier)**

**Results:**  4060

**Note:** Searched Embase and Embase classic only.

| Step |  |
| --- | --- |
| 1 | sepsis/mj OR (septic* OR sepsis OR septic$emia OR 'systemic inflammatory response syndrome' OR py$emia):ti,ab |
| 2 | 'mass screening'/de OR (screen* NEAR/3 (manual* OR tool*)):ti,ab OR (identif* OR recogni* OR diagnos* OR screen* OR detect* OR surveill* OR monitor*):ti |
| 3 | Hospital/exp OR hospital*:ti,ab OR 'hospital emergency service'/de OR 'emergency department*':ti,ab OR 'intensive care unit'/exp OR 'intensive care unit*':ti,ab OR ICU:ti,ab OR ((moderate OR medium OR intermediate) NEAR/5 (unit OR units OR ward OR wards)):ti,ab |
| 4 | (1 and 2 and 3) |
| 5 | 'animal'/exp NOT 'human'/de |
| 6 | 4 NOT 5 |
| 7 | 6 AND ([embase]/lim NOT ([embase]/lim AND [medline]/lim) OR ([embase classic]/lim NOT ([embase classic]/lim AND [medline]/lim))) |
| 8 | 7 AND (2000:py OR 2001:py OR 2002:py OR 2003:py OR 2004:py OR 2005:py OR 2006:py OR 2007:py OR 2008:py OR 2009:py OR 2010:py OR 2011:py OR 2012:py OR 2013:py OR 2014:py OR 2015:py OR 2016:py OR 2017:py OR 2018:py OR 2019:py OR 2020:py OR 2021:py OR 2022:py OR 2023:py OR 2024:py) |
| 9 | English:la |
| 10 | 8 and 9 |

**CINAHL Complete (EBSCOhost)**

**Results:**  1655

| Step |  |
| --- | --- |
| 1 | (MM  sepsis+) OR ((TI septic* OR AB septic*) OR (TI sepsis OR AB sepsis) OR (TI septic#emia OR AB septic#emia) OR (TI "systemic inflammatory response syndrome" OR AB "systemic inflammatory response syndrome") OR (TI py#emia OR AB py#emia)) |
| 2 | (MH "health screening") OR ((TI screen* OR AB screen*) N3 ((TI manual* OR AB manual*) OR (TI tool* OR AB tool*))) OR ((TI identif*) OR (TI recogni*) OR (TI diagnos*) OR (TI screen*) OR (TI detect*) OR (TI surveill*) OR (TI monitor*)) |
| 3 | (MH Hospitals+) OR (TI hospital* OR AB hospital*) OR (MH "emergency service+") OR (TI "emergency department*" OR AB "emergency department*") OR (MH "intensive care units+") OR (TI "intensive care unit*" OR AB "intensive care unit*") OR (TI ICU OR AB ICU) OR (((TI moderate OR AB moderate) OR (TI medium OR AB medium) OR (TI intermediate OR AB intermediate)) N5 ((TI unit OR AB unit) OR (TI units OR AB units) OR (TI ward OR AB ward) OR (TI wards OR AB wards))) |
| 4 | (1 and 2 and 3) |
| 5 | (MH animals+) NOT (MH human) |
| 6 | (4 NOT 5) |

**Supplemental Table e1: Screening tools used**

| **Author, year** | | **Screen used** | **Vitals** | | | | **Labs** | | **Assessment** | | | |  |
| --- | --- | --- | --- | --- | --- | --- | --- | --- | --- | --- | --- | --- | --- |
|  |  |  | **SBP/MAP** | **HR** | **RR** | **Temperature** | **WBC** | **Lactate** | **AMS** | **O2 req.** | **UOP** | **Suspected infection** | **Other screening elements** |
| Emergency Department (ED) | | | | | | | | | | | | | |
|  | Bader, 2020 | qSOFA | x |  | x |  |  |  | x |  |  |  |  |
|  | Hart, 2017 |  | x | x | x | x |  |  | x |  | x |  |  |
|  | Idrees, 2016 | SEAT | x | x | x | x |  | x | x | x |  | x | Sepsis risk factors (e.g. hospitalization, indwelling device, age,) |
|  | McDonald, 2018 | SIRS + |  | x | x | x | x |  |  |  |  | x | Sepsis risk factors (e.g. hospitalization, indwelling device, chemotherapy) |
|  | Patocka, 2014 |  | x | x | x |  |  |  | x | x |  |  | Hyperglycemia, mottled skin |
|  | Shah, 2018 |  | x | x | x | x |  |  | x | x |  | x | Rigors |
|  | Song, 2019 | SOFA | x |  |  |  |  |  | x |  | x |  | P/F ratio, platelet count, bilirubin, creatinine |
|  | Suttapanit, 2022 | REWs | x | x | x | x |  |  | x | x |  |  |  |
|  | Tedesco, 2017 | SIRS |  | x | x | x | x |  |  |  |  |  |  |
| Hospital ward | | | | | | | | | | | | | |
|  | Alberto, 2020 | qSOFA | x |  | x |  |  |  | x |  |  |  |  |
|  | Jones, 2015 |  |  | x | x | x | x |  | x |  |  |  |  |
|  | Roney, 2019 | MEWS | x | x | x | x | x | x | x | x | x |  |  |
|  | Torsvik, 2016 | SIRS + | x | x | x | x | x | x | x | x | x |  | Platelet count |
| Intensive Care Unit (ICU) | | | | | | | | | | | | | |
|  | Croft, 2014 | MEWS | x | x | x | x | x | x | x | x | x |  |  |
|  | Moore, 2009 | SIRS |  | x | x | x | x |  |  |  |  |  |  |
|  | Rincon, 2011 |  |  |  |  |  |  |  |  |  |  |  | “Tele-ICU coordinated with the bedside staff to collect the information” |
| ED, hospital ward, and ICU | | | | | | | | | | | | | |
|  | Westphal, 2011 |  | x | x | x | x |  |  | x | x | x |  |  |
| SOFA = sequential organ failure assessment; qSOFA = quick SOFA; SIRS = systemic inflammatory response syndrome; SEAT = sepsis early alert tool; REW = Ramathibodi Early Warning score; SBP/MAP = systolic blood pressure / mean arterial pressure; HR = heart rate; RR = respiratory rate; WBC = white blood count; AMS = altered mental status; O2 req. = supplemental oxygen requirement; UOP = urine output; P/F ratio = PaO2/FiO2 ratio. EHR sepsis indicator = electronic health record indicator (banner, flag, etc) to visually alert clinicians that the patent has/may have sepsis. | | | | | | | | | | | | | |

**Supplemental Table e2: Methods used to identify sepsis cases**

| **Author, year** | | **Sep-3*** | **Sepsis, severe sepsis, and/or septic shock**** | **Diagnosis codes** | | **Other criteria used and implementation details** |
| --- | --- | --- | --- | --- | --- | --- |
|  |  |  |  | **ICD-9** | **ICD-10** |  |
| Emergency Department (ED) | | | | | | |
|  | Bader, 2020 |  |  |  |  | Not reported |
|  | Hart, 2017 |  |  |  | x |  |
|  | Idrees, 2016 |  | x |  |  | Implemented via single physician retrospective chart review |
|  | McDonald, 2018 |  | x |  |  | SIRS + suspected infection OR sepsis risk factors. Unclear how this was implemented |
|  | Patocka, 2014 |  |  | x |  | Admission/discharge diagnoses + ED death certificate |
|  | Shah, 2018 |  |  | x |  |  |
|  | Song, 2019 | x |  |  |  | Retrospective chart review, unclear who performed review |
|  | Suttapanit, 2022 | x |  |  |  | Unclear how this was implemented |
|  | Tedesco, 2017 |  |  |  | x |  |
| Hospital ward | | | | | | |
|  | Alberto, 2020 | x |  |  |  | Implemented via single intensivist retrospective chart review |
|  | Jones, 2015 |  |  | x |  |  |
|  | Roney, 2019 |  |  |  |  | Not reported |
|  | Torsvik, 2016 |  |  |  |  | Positive blood culture |
| Intensive Care Unit (ICU) | | | | | | |
|  | Croft, 2014 |  | x |  |  | Prospective enrollment by researchers, then cases screened by committee |
|  | Moore, 2009 |  |  | x |  |  |
|  | Rincon, 2011 |  |  |  |  | Not reported |
| ED, hospital ward, and ICU | | | | | | |
|  | Westphal, 2011 |  | x |  |  | Unclear how this was implemented |
| *Sep-3 definition: Based on 2016 International Consensus Definition of sepsis = life-threatening organ dysfunction caused by dysregulated host response to infection.  **Any combination of Sepsis = 2 or more SIRS criteria + suspected infection, Severe Sepsis = sepsis + organ dysfunction OR lactate >4.0 mmol/L, Septic shock = severe sepsis + hypotension (SBP<90 or MAP<70) after fluid bolus. | | | | | | |

**Supplemental Table e3: Processes of care and mortality outcomes by study**

| Author, year | | Time to antibiotics | | Time to lactate or blood cultures, OR lactate measured | | Time to IV fluids | | Sepsis bundle compliance | | Mortality | | |
| --- | --- | --- | --- | --- | --- | --- | --- | --- | --- | --- | --- | --- |
|  |  | Improved | No change | Improved | No change | Improved | No change | Improved | No change | Improved | No change | Numeric decrease: not significant or significance not reported |
| Emergency Department (ED) | | | | | | | | | | | | |
|  | Bader, 2020 | x |  | x |  | x |  |  |  | x |  |  |
|  | Hart, 2017 |  | x |  | x |  |  |  |  |  |  |  |
|  | Idrees, 2016 | x |  |  |  |  |  |  |  |  |  |  |
|  | McDonald, 2018 | x |  | x |  | x |  |  |  |  |  | x |
|  | Patocka, 2014 | x |  | x |  |  | x |  |  |  |  | x |
|  | Shah, 2018 | x |  |  |  |  |  |  | x |  | x |  |
|  | Song, 2019 |  | x |  |  |  |  | x |  | x |  |  |
|  | Suttapanit, 2022 |  | x |  |  |  |  |  |  |  | x |  |
|  | Tedesco, 2017 |  |  |  |  |  |  |  |  | x |  |  |
| Hospital ward | | | | | | | | | | | | |
|  | Alberto, 2020 |  |  |  |  |  |  |  | x |  |  |  |
|  | Jones, 2015 |  |  |  |  |  |  |  |  | x |  |  |
|  | Roney, 2019 |  |  |  |  |  |  |  |  |  |  | x |
|  | Torsvik, 2016 |  | x |  |  |  |  |  |  | x |  |  |
| Intensive Care Unit (ICU) | | | | | | | | | | | | |
|  | Croft, 2014 |  | x |  |  |  |  |  |  |  | x |  |
|  | Moore, 2009 |  |  |  |  |  |  |  |  |  |  | x |
|  | Rincon, 2011 | x |  | x |  | x |  |  |  |  |  |  |
| ED, hospital ward, and ICU | | | | | | | | | | | | |
|  | Westphal, 2011 |  |  |  |  |  |  |  | x | x |  |  |
| Total # of studies | | 6 | 5 | 4 | 1 | 3 | 1 | 1 | 3 | 6 | 3 | 4 |

**Supplemental Table e4: Outcomes on screening adherence and screening tool test characteristics**

| **Author, year** | **Adherence** | **Test characteristics** |
| --- | --- | --- |
| Emergency Department | | |
| Patocka, 2014 | 64% of eligible patients had screening performed |  |
| Hospital Ward | | |
| Alberto, 2020 | 92.5% of patients were screened a median (IQR) of 70.9% (27%) of the possible times. Nurses activated sepsis alerts in 21/64 (32.8%) patients with positive screens, and delays of ≥7 h were observed for most patients with only five alerts communicated immediately |  |
| Jones, 2015 | 10% of eligible patients were screened in the first year of the study, increased to 33% of patients in the final year |  |
| Intensive Care Unit | | |
| Croft, 2014 |  | 683 positive screens, 201 confirmed sepsis cases = PPV 29.4% |
| Moore, 2009 |  | sensitivity 96.5%, specificity 96.7%, PPV 80.2%, NPV 99.5% |
| Rincon, 2011 | At least 85% of patients were screened at least 1 time per admission |  |

**Supplemental Table e5: ROBINS-I risk of bias assessment**


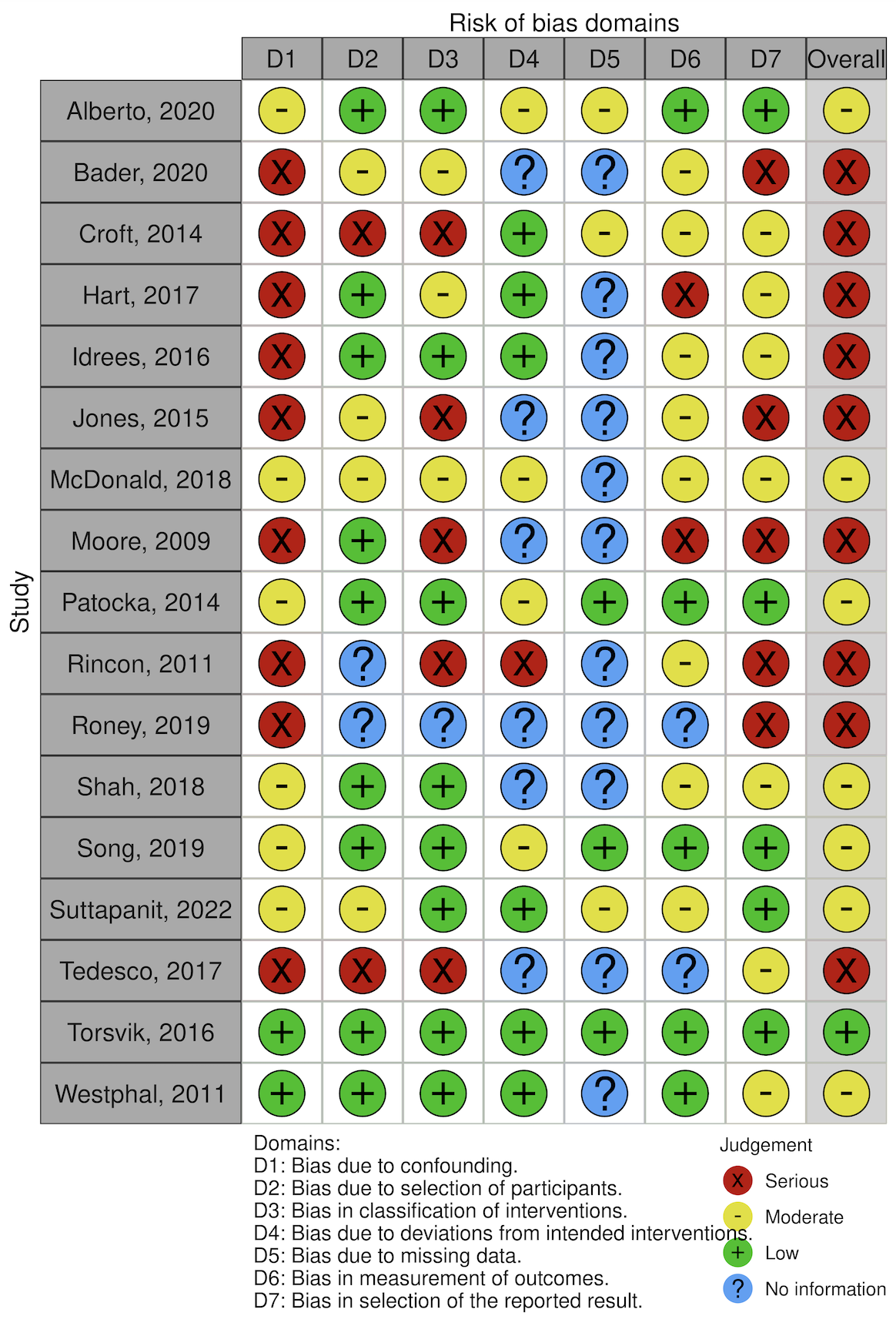

Supplement: Supplementary file 1 — SepScreenRev_R&R_Supplement_2025.10.23. [file JHM-21-539-s001.docx]
